# G6PD deficiency in male individuals infected by *Plasmodium vivax* malaria in the Brazilian Amazon: a cost study

**DOI:** 10.1186/s12936-015-0647-x

**Published:** 2015-03-24

**Authors:** Henry M Peixoto, Marcelo AM Brito, Gustavo AS Romero, Wuelton M Monteiro, Marcus VG de Lacerda, Maria Regina F de Oliveira

**Affiliations:** Centre for Tropical Medicine, University of Brasília, Brasília, Federal District Brazil; University Centre of Brasília, Brasília, Federal District Brazil; Tropical Medicine Foundation Dr. Heitor Vieira Dourado, Manaus, Amazonas Brazil; University of the State of Amazonas, Manaus, Amazonas Brazil; National Institute for Science and Technology for Health Technology Assessment (IATS/CNPq), Porto Alegre, RS Brazil; Centro de Pesquisas Leônidas & Maria Deane, FIOCRUZ, Manaus, Amazonas Brazil

**Keywords:** Glucose-6-phosphate dehydrogenase deficiency, Primaquine, Haemolysis, Malaria, *Plasmodium vivax*, Economic analysis

## Abstract

**Background:**

Deficiency of the enzyme G6PD (G6PDd) is caused by mutations in the gene G6PD, which plays an important role in protecting the red blood cell against oxidizing agents; it is linked to chromosome X, and it may affects both sexes. The clinically relevant manifestations, such as acute haemolytic anaemia, mainly occur in men, however. The 8-aminoquinoline primaquine, which is the medication used in the radical treatment of malaria caused by *Plasmodium vivax,* represents the main factor that triggers complications associated with G6PDd. The current study aims to estimate the costs of G6PDd among male individuals infected by *P. vivax* in the Brazilian Amazon.

**Methods:**

This is an economic analysis developed within the Brazilian National Health System perspective for the years of 2009, 2010 and 2011. Direct medical and non-medical costs were estimated for G6PDd in the Brazilian Amazon, considering among those suffering from the deficiency the costs of diagnosing infection by *P. vivax*, its treatment and severe adverse events that require hospitalization and were connected to the use of primaquine.

**Results:**

The estimates of the average costs of diagnosing vivax malaria*,* of its treatment and of severe adverse events after using primaquine among the carriers of G6PDd, over the three evaluated years, corresponded to US$ 739,410.42; US$ 2,120.04 and US$ 4,858,108.87, respectively. The results indicate that the average total cost in the study period corresponded to US$ 5,599,639.33, varying in accordance with the sensitivity analysis between US$ 4,439,512.14 and US$ 6,702,619.24.

**Conclusion:**

The results indicate that the use of primaquine among men with G6PDd who are infected by *P. vivax* represents a heavy burden on the public health service of Brazil.

**Electronic supplementary material:**

The online version of this article (doi:10.1186/s12936-015-0647-x) contains supplementary material, which is available to authorized users.

## Background

The enzyme glucose-6-phosphate dehydrogenase (G6PD) exerts an important role in protecting the red blood cell from oxidizing agents, being fundamental for the neutralization of the toxic products of oxygen originating principally from oxidizing drugs and from infections, which can damage the erythrocyte membrane, resulting in haemolysis [1]. G6PD deficiency (G6PDd) is a genetic disease caused by mutations in the gene that codifies the enzyme. Currently, 186 mutations are known that produce various functional manifestations, both biochemical ones and phenotypical ones [2,3]. The gene for G6PD is situated in the telomeric region of the long arm of the X sex chromosome (band Xq28), and it can affect both sexes, but the clinically important manifestations occur mainly in male individuals [4,5]. In Latin America, primaquine (PQ), an 8-aminoquinoline used in the radical treatment of the infection (against liver hypnozoites) caused by *Plasmodium vivax,* represents the main agent associated with severe haemolysis, imposing life-threatening risks on patients with G6PDd [5,6].

Cases of G6PDd are widely distributed in countries considered endemic for malaria, with an estimate of 353 million people affected, constituting a general prevalence of 8%, and of whom 220 million are men [7]. A greater prevalence is observed in some regions of Sub-Saharan Africa, followed by Asia and Latin America [7]. In Brazil, the nine states that compose the Brazilian Amazon concentrate more than 99% of the cases of malaria diagnosed, with 900,000 new cases having been reported between 2009 and 2011, of which 85% are *P. vivax* malaria [8]. In this region, the estimated prevalence of G6PDd or the male sex is 4.5%, with a predominance of the African variant [9]. Various cases of haemolysis induced by PQ have been reported in the Brazilian Amazon. The problem should be dealt with by health authorities [5,10], principally at this moment in which regional and international efforts are being harnessed to reduce mortality caused by malaria [11]. In infections caused by *P. vivax,* most parasites develop and rapidly leave the liver, but some, named hypnozoites, remain latent in the hepatocytes and will later cause a relapse [12,13]. In this context, PQ is currently the only licensed drug that is capable of combating hypnozoites [13,14].

In Brazil, the public health service, oriented by the National Program for the Prevention and Control of Malaria [Programa Nacional de Prevenção e Controle da Malária (PNCM)], is responsible for the diagnosis and treatment of malaria throughout the whole country. The PNCM does not include testing for G6PDd in its routine, and it recommends the use of PQ for all patients infected by *P. vivax*, except for pregnant women and children under six months of age [12]. In this respect, studies indicate that the use of an 8-aminoquinoline for someone bearing G6PDd may result in severe anaemia, kidney failure and death as a result of haemolysis [4,15]. Thus, as health professionals do not know if the individuals being treated with PQ are or are not G6PDd, some bearers of the deficiency receive the medication and develop complications, generating a resulting demand for resources from the public health system.

In Latin America, the adoption of public policies focused on the male population may be more efficient in reducing haemolysis induced by PQ because the cases in females are rare [5]. However, due to the difficulty of obtaining information, especially related to hospitalization, there are no studies in the literature that evaluate the cost of G6PDd regarding strategies that recommend the use of PQ in treating vivax malaria. This knowledge is essential as a basis for discussions related to the adoption of safer and more effective public policies for malaria control in Brazil. The present study estimated for the first time the costs of G6PDd among male individuals infected by *P. vivax* in the Brazilian Amazon.

## Methods

### Study design

This is an economic analysis developed within the Brazilian National Health Service perspective (Sistema Único de Saúde - SUS) for the years of 2009, 2010 and 2011. Direct medical and non-medical costs were estimated for G6PDd for the Brazilian Amazon, considering the male individuals suffering from malaria and carrying G6PDd. Costs were calculated for the diagnosis and treatment of vivax malaria and for the severe adverse events associated with the use of PQ. The costs were obtained in the Brazilian currency (the real, R$) and converted into American dollars (US$), using the mean exchange rate for the years under study.

The Brazilian Amazon is composed of 775 municipalities distributed in nine states (Acre, Amapá, Amazonas, Maranhão, Mato Grosso, Tocantins, Rondônia, Roraima and Pará), corresponding to an area of land of 5,109,812 km^2^, covering about 60% of Brazilian territory. In the region there are about 21 million inhabitants, with a demographic density of 4.14 inhabitants/km^2^, which is considered the lowest in Brazil [16]. In the years evaluated, on average 502 municipalities reported cases of vivax malaria among male individuals [8].

### Assumptions for the costs of G6PDd among male individuals infected by *P. vivax* in the Brazilian Amazon, 2009, 2010 and 2011

Diagnosis and treatment of vivax malaria among carriers of G6PDd:

- Diagnosis of vivax malaria**:** performed by blood microscopy test, using the Walker method of thick blood smear;
- Microscope and microscope maintenance: considered as one microscope and one maintenance per year per reporting unit, assuming that all reporting units identified in Information System for Epidemiological Surveillance of Malaria (SIVEP/MALÁRIA) provided the diagnosis of *P. vivax* malaria;
- Training of microscopists: one training period per year per municipality that reported case of vivax malaria among men;
- Health worker: the cost of diagnosis and treatment considered the health agent involved in active demand of new cases and the microscopist involved in providing the diagnosis and medication. The monthly salary of the microscopist and health worker were based on the salary of Amazonas State, both relating to the auxiliary level career with workload of 40 hours per week.

Deficiency of G6PD among men submitted to treatment with PQ:

- Costs of specialized medical appointment: one medical appointment specialized in admission and another after hospital discharge.
- Tests performed before and after hospitalization: all carrying G6PDd with severe adverse events and requiring hospital care were submitted to pre-admission tests. Among them, the patients released from hospital did further tests during follow-up monitoring.
- Hospital costs, where all patients with severe adverse effects were hospitalized:
- Professional costs were calculated based on time spent per patient/day per doctor, nurse and nursing technician, assuming the average salaries in Amazonas state;
- Hospital food: six meals a day for each patient, assuming values of the FMT-HVD, Amazonas State.

### Definition of cases and sources of information

Because Brazil does not carry out the test for G6PDd before starting treatment with PQ [17], as a basis for defining cases we took the routine implemented at the Dr. Heitor Vieira Dourado Tropical Medicine Foundation (FMT-HVD), a reference in malaria treatment and research in Brazil, and fully funded by SUS. It is situated in Manaus, the capital of Amazonas state, and in 2013 was responsible for dealing with 2,315 cases of malaria, which represent 31.85% of the 7,268 cases reported in Manaus [8]. The FMT-HVD emphasizes that patients should return to the health centre if they develop choluria, fever and jaundice, besides serving as a tertiary care centre to where complicated cases from all over the municipality are referred [18]. In summary, number of admissions in this centre is a close proxy of all cases requiring hospitalization due to severe haemolysis in Manaus and surrounding areas. Thus, based on the routine described, the costs were calculated from the following definition for a case: male carriers of G6PDd who asked for diagnosis of malaria; were diagnosed as new cases of vivax malaria; used treatment of PQ and evolved symptoms compatible with a severe adverse events resulting from treatment. Adverse events considered severe included individuals who needed hospitalization, corroborating the definition presented in a recent review published by WHO [19]. The epidemiological parameters considered are presented in Table 1.

**Table 1:** **Epidemiological parameters in the Brazilian Amazon, 2009, 2010 and 2011**

**Items**	**Years**			
	**2009**	**2010**	**2011**	**Sources**
Number of males submitted to thick blood smear test in Brazilian Amazon^1^	1,596,725	1,643,714	1,781,812	[8]
Number of males with G6PDd submitted to thick blood smear test in Brazilian Amazon^2^	71,853	73,967	80,182	[8] and [9]
Number of males submitted to thick blood smear test in Brazilian Amazon by active demand^3^	878,473	903,808	968,145	[8]
Number of males with G6PDd submitted to thick blood smear test in Brazilian Amazon by active demand^4^	39,531	40,671	43,567	[8,9]
Number of vivax malaria cases among males	155,413	173,559	141,803	[8]
Number of males with G6PDd among those diagnosed with *P. vivax* ^5^	6,994	7,810	6,381	[8,9]
Number of units that diagnosed cases of vivax malaria	1,814	1,797	1,639	[8]
Number of municipalities in Brazilian Amazon that reported cases of vivax malaria among men	524	511	472	[8]
Number of males hospitalized with G6PDd after treatment with PQ^6^	6,594	7,364	6,016	[8-10]
Number of deaths attributed to the use of PQ among carrier men of G6PDd ^7^	189	211	172	[8-10]

^1^Number estimated from multiplying the total number of people of both genders, subject to examination, by the proportion of males.

^2^Number estimated from the number of males submitted to thick blood smear examination in Brazilian Amazon weighted by prevalence of G6PDd for males (4.5%).

^3^Number obtained from multiplying the estimate of males submitted to thick blood smear test by the overall proportion of individuals diagnosed by active demand.

^4^Number estimated from multiplying the number of males submitted to thick blood smear test in Brazilian Amazon by active demand weighted by the prevalence of G6PDd for males (4.5%).

^5^Number estimated from the total cases of *P. vivax* malaria among males in Brazilian Amazon weighted by prevalence of G6PDd for males (4.5%).

^6^Number estimated from the number of males with G6PDd among those diagnosed with *P. vivax* multiplied by the proportion of hospitalizations (94.29%) among males with G6PDd monitored from 2009 to 2011 in the FMT-HVD.

^7^Number estimated from the number of males hospitalized with G6PDd after treatment with PQ, multiplied by the proportion of deaths (2.86%) among the bearers of the deficiency monitored in the FMT-HVD.

The economic data that supported the estimate of costs (Table 2) were identified in the scientific literature, in the System for Managing Standard Prices for Procedures, Medicines and Materials within SUS (SIGTAP) [20], in the Bank of Prices in Health (BPS) within the Ministry of Health (MoH) [21], in documents and databanks supplied by the Brazilian MoH and by the FMT-HVD. The values not identified in the evaluated years were adjusted for the years of 2009, 2010 and 2011, based on the official rate of inflation estimated by the accumulated National Consumer Price Index (IPCA) [22]. Epidemiological data and service data were obtained from the SIVEP/MALÁRIA [8], from the literature (Table 1) and from unpublished information obtained in the documents and databanks of FMT-HVD.

**Table 2:** **Costs of G6PDd in males infected by** ***P. vivax*** **in the Brazilian Amazon, 2009, 2010 and 2011**

**Items**	**Reference values - base-case (US$)/year**						**Sources**
**A - Diagnosis of vivax malaria**	**2009**		**2010**		**2011**		
	**All males**	**Males with G6PDd** ^**1**^	**All males**	**Males with G6PDd** ^**1**^	**All males**	**Males with G6PDd** ^**1**^	
- Thick blood smear	1,814,409.17	81,648.41	2,202,950.33	99,132.77	2,665,142.63	119,931.42	[24]
- Microscope	1,096,068.22	49,323.07	1,280,603.64	57,627.16	1,303,580.23	58,661.11	[25]
- Microscope maintenance	83,204.59	3,744.21	97,213.02	4,374.59	98,957.22	4,453.08	[30]
- Health workers	3,912,989.04	176,084.51	4,754,380.68	213,947.13	5,889,094.46	265,009.25	[31]
- Annual training (microscopist)	7,223,201.42	325,044.07	8,307,793.49	373,850.70	8,564,439.54	385,399.78	[30]
- Total costs (A)	14,129,872.43	635,844.27	16,642.941.17	748,932.35	18,521,214.09	833,454.64	
**B – Drug treatment for** ***P. vivax***	**2009**		**2010**		**2011**		
	**All males**	**Males with G6PDd**	**All males**	**Males with G6PDd**	**All males**	**Males with G6PDd**	
- Therapeutic scheme	41,284.62	1,857.92	58,635.90	2,638.56	41,415.28	1,863.65	[32]
**C – Assistance to carriers of G6PDd treated with PQ**	**2009**		**2010**		**2011**		
	**Males with G6PDd**		**Males with G6PDd**		**Males with G6PDd**		
- Pre-admission tests	74,659.04		94,701.04		82,037.35		[20,24]
- Medical appointments	66,271.36		83,681.82		72,047.90		[20]
- Hospitalization^2^	3,530,482.47		4,457,992.22		3,838,217.22		[20]
- Hospital food	432,757.87		580,711.73		538,733.13		[33]
- Health workers	141,826.64		188,506.44		175,245.56		[34]
- Tests performed after hospitalization	64,235.79		81,530.89		70,688.14		[20,24]
- Total costs (C)	4,310,233.17		5,487,124.14		4,776,969.30		
- Total costs (A + B + C)^3^	4,947,935.36		6,238,695.05		5,612,287.59		

^1^Weighted by the prevalence of G6PDd (4.5%).

^2^In the years evaluated, 19% and 81% of the costs corresponded to hospitalization in ICT and ward, respectively.

^3^Males with G6PDd.

### Estimate of costs

#### Diagnosis and treatment of vivax malaria among carriers of G6PDd

The diagnosis of malaria in Brazil is done by blood microscopy test, using the Walker method of thick blood smear, as recommended by the MoH [23]. The costs considered in the diagnostic strategy for vivax malaria included materials used in the thick blood smear, microscopes, maintenance of microscopes, salaries of microscopists and of the community health agents (CHA) involved in the diagnosis carried out as active demand and in the training of microscopists.

The cost of a thick blood smear used in the base case was estimated from the study carried out by Macauley [24] in the Brazilian Amazon. The costs obtained were multiplied by the estimate of the number of G6PDd carriers who did the test [8], making it possible to obtain the total cost of the test in accordance with the year under evaluation.

The cost of a microscope, estimated at US$ 6,272.00 and presented by Oliveira [25], obtained from unpublished information from the Secretariat of Health Surveillance of the MoH (SHS/ MoH), was used by all the units that provided malaria case reports. The annual equipment cost for the base case was calculated for a period of use of 15 years [26], using a discounting rate of 5% [27,28] and annualization factor of 10.380 [29]. The maintenance costs for one microscope, estimated to be US$ 46.00, were based on the cost reported by Oliveira [30], obtained from information from the Malaria Laboratory at the Evandro Chagas Institute, SHS/MoH. The total costs of the microscope and its maintenance were obtained by the product of the unit value multiplied by the number of units that diagnosed cases of vivax malaria, in accordance with the year being considered [8]. At the end, the cost was weighted by the prevalence of G6PDd [9].

The costs of the salaries for the microscopists and the health agents were calculated based on a document from the WHO [31]. This stratified the risk, expressed by the proportion of positive slides, per Amazon state [8]. It allowed us to obtain the number of slides carried out per professional per day, the number of professionals needed and the total salary by risk stratum, the sum of which corresponded to the total cost. Costing on salaries for microscopists considered all the male individuals submitted to the test and costing on health agents only considered those submitted to diagnosis by active demand [8], given that this professional essentially works with this strategy. Finally, the salary costs were weighted by prevalence of G6PDd [9].

The average cost for training the microscopist was obtained from the study published by Oliveira *et al.* [30], which used the cost of one training period a year, lasting 40 hours, based on data supplied by Brazilian MoH and coming from training given to microscopists in the Brazilian Amazon in 2006 [30]. The cost for all training periods represents the product of the number of training periods, considering one per year per municipality that reported a case of vivax malaria among men, in the year referred to. The final value was weighted by prevalence of G6PDd [9].

The cost of anti-malarial medicines was based on the therapeutic scheme stipulated by the MoH (chloroquine for three days and PQ for seven days) [12] and informed by the Strategic Input Information System of the SHS/MoH [32] as a cost of a tablet of PQ for children (5 mg), of PQ for adults (15 mg) and of chloroquine 150 mg. Next, the cost of the therapeutic scheme was calculated, and this was weighted by the proportion of individuals in the age categories proposed by therapeutic scheme [12]. The total cost with treatment was calculated by multiplying the cost of the therapeutic scheme and the estimate for total number of cases of vivax malaria in male carriers, in accordance with the year being evaluated.

#### Deficiency of G6PD among men infected by *Plasmodium vivax* submitted to treatment with PQ

The cost of G6PDd among carriers of *P. vivax* submitted to treatment with PQ considered the individual costs of the specialist medical consultation and laboratory tests before hospitalization and after release, expenditure on hospital care, health professionals and hospital food. The total costs of specialized consultations and of laboratory tests were estimated considering the number of patients hospitalized and the number of patients who were released from hospital in the years under research. The cost of a specialized medical consultation was obtained in SIGTAP [20] and did not undergo variations in the period of this study. Both the set of pre-admission tests and those carried out after release from hospital were proposed based on the opinion of specialists at FMT-HVD (see the next subsection) and identified in SIGTAP [20], except in the case of the thick blood smear which, as it does not appear in SIGTAP, was obtained in the literature. The quantitative test for G6PD was included in the set of tests carried out after hospitalization, with the aim of confirming the deficiency and supporting the prevention of new severe adverse events.

The values of hospital care costs paid for by the SUS, by means of the Authorization for Hospitalization (AIH), were obtained from SIGTAP [20], considering the hospitalizations in the ward for treatment of anaemia due to G6PDd (CID 10 – D550) and daily costs in the Intensive Care Unit (ICU), both paediatric (code 080201007–5) and adult (code 080201009–1). The component corresponding to professional services was subtracted from the total values of the daily hospital costs. To calculate the cost of hospitalization coming from payment of the AIHs, the daily value was multiplied by the average 4.25 days in the ward (hospitalization for anaemia due to G6PDd) and the 7.5 days in the ICU (paediatric and adult, both bearing the same daily cost, according to SIGTAP [20]) [10], and weighted by the proportion of individuals hospitalized in the ward (97%) and in the ICU (6%), observing that 3% of the individuals were first in the ward and later needed the ICU [10]. The total cost of hospitalization represents the product of multiplying the estimated cost of hospitalization and the estimated number of hospitalized patients.

The cost of hospital food considered that all hospitalized individuals received the following meals: breakfast, snack, lunch, snack, dinner and light supper. The calculation of the cost per patient was obtained by means of multiplying the cost of a meal, obtained from the chart of prices paid by the FMT-HVD [33], by the average number of days spent in hospital, followed by summing the costs per meal.

The salary costs related to hospitalization due to G6PDd were based on the average salaries in the state of Amazonas for a doctor, nurse and nursing technician [34]. Using the monthly salary, the value of an hour’s work was calculated, and this was multiplied by the estimated number of hours dedicated to the patient, obtained from data supplied by the FMT-HVD. The result was weighted by the proportion of patients hospitalized by G6PDd among those hospitalized in the FMT-HVD, during the years under study.

#### Tests performed in carriers of G6PDd with severe adverse events associated with the use of primaquina

- Pre-admission tests: thick blood smear, complete blood count, levels of: serum creatinine, blood urea nitrogen, SGOT and SGPT, total bilirubin and fractions, complete urinalysis and reticulocyte count.
- Tests carried out after release from hospital: thick blood smear, complete blood count, levels of: serum creatinine, blood urea nitrogen, SGOT and SGPT, total bilirubin and fractions, complete urinalysis and testing for G6PDd.

### Analysis of sensitivity

Individual variation of all the costs was carried out, with the exception of the cost of specialist medical consultations and the cost of the therapeutic scheme used in the treatment of vivax malaria, since there were no variations for medication and consultation over the evaluated years [20,32]. The values used for the variations were estimated using information that came from the literature or by variation of 20% above and below the base case.

## Results

Table 1 contains the epidemiological and service data that formed the basis for costing on G6PDd among male individuals infected with *P. vivax* in the Brazilian Amazon. During the evaluated period, in fact, no single woman was hospitalized due to haemolysis following malaria treatment. According to estimates presented in Table 1, the strategy for using PQ against *P. vivax* [12] is considered to have treated 21,185 male carriers of G6PDd between the years of 2009 and 2011, leading to 19,974 hospitalizations and 572 deaths.

**Figure 1:**
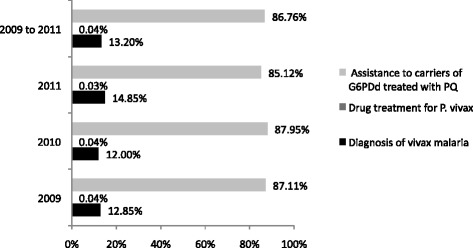
**Relative costs of G6PDd in males infected by** ***P. vivax*** **in the Brazilian Amazon, 2009, 2010, 2011.**

Costing on G6PDd among men infected by *P. vivax* (Table 2) indicates that the cost of diagnosing *P. vivax* varied between US$ 635,844.27 (2009) and US$ 833,454.64 (2011); the cost of medicinal treatment of *P. vivax* varied from US$ 1,857.92 (2009) to US$ 2,638.56 (2010); and the cost of severe adverse events associated with the use of PQ among carriers of G6PDd, considering the tests carried out before and after hospitalization, medical consultations and hospitalizations, varied from US$ 4,310,233.17 (2009) to US$ 5,487,124.14 (2010). For all the evaluated years, the relative cost provided in Figure 1 indicates the predominance of costs arising from the assistance to carriers of G6PD treated with PQ. The detailed analysis of sensitivity of the costs can be seen in Additional file 1.

In Table 3, the average costs are presented for the base case and the variations considered for the three years, for the same items previously cited (Table 2), as well as the total cost reached by adding up all the items. The data indicate that the average cost of G6PDd, over the three evaluated years, corresponded to US$ 5,599,639.33, varying in accordance with the analysis of sensitivity between US$ 4,439,512.14 and US$ 6,702,619.24.

**Table 3:** **Average cost of G6PDd in male individuals infected with** ***P. vivax*** **and variations in the Brazilian Amazon, 2009, 2010 and 2011**

**Items**	**Base-case (US$)**	**Lower limit (US$)**	**Upper limit (US$)**
Diagnosis of vivax malaria^1^	739,410.42	532,081.82	920,943.11
Therapeutic scheme^1^	2,120.04	2,120.04	2,120.04
Assistance to carriers of G6PDd treated with PQ^1^	4,858,108.87	3,905,310.28	5,779,556.08
Total cost	**5,599,639.33**	**4,439,512.14**	**6,702,619.24**

^1^The values in the base case and variations represent the average costs in the years considered in the study.

## Discussion

Malaria represents a severe public health problem in Brazil, being associated with socio-economic problems and failures in preventive measures and social protection actions [35,36]. It presents probable repercussions in the disease-impoverishment-poverty cycle, and is thus considered one of the diseases associated with poverty worldwide, impacting negatively on family income, which then further increases the vulnerability of the affected populations [37,38]. In recent years, the Brazilian Amazon has presented a sharp decline in the incidence of malaria by *P. falciparum*; however, the number of people infected by *P. vivax* has not fallen at the same rate [35], perhaps because the parasite is able to provoke relapses weeks or months after the initial infection [39]. Between the years 2009 and 2011, 767,000 cases of vivax malaria were reported, of which 470,000 occurred in male individuals, affecting mainly men of productive age [8]. However, the female population and those in other age categories also suffer from it, especially children between five and 14 years of age, who may do badly at school due to malaria, thus leading to negative economic impacts and a drop in productivity in the long-term [40].

The high number of cases of vivax malaria is particularly important because PQ has been adopted as the strategy for effecting a radical cure of vivax malaria in Brazil, increasing the risk of severe adverse events and of deaths, since the use of PQ at a dose of 0.5 mg/kg/day, for seven days, may cause acute haemolysis among carriers of G6PDd [5,41]. Although the estimates related to the incidence of severe adverse events and to deaths associated with the use of PQ continue to be imprecise [19], evidence obtained from campaigns that used mass treatment in Azerbaijan, Afghanistan, Tajikistan and Korea demonstrates that rigorous management of the risks may minimize their effects on populations with various degrees of G6PDd [42].

The number of hospitalizations and of deaths presented was calculated from the proportion of hospitalizations (94.29%) and of deaths (2.86%) among the carriers of G6PDd attended in the FMT-HVD [10]. Thus, both proportions represent the situation in a reference centre for treatment, and this may differ from what happens in remote areas and small municipalities in the Brazilian Amazon. In the latter case, the proportion of hospitalizations may have been over-estimated and the deaths under-estimated, given that not only are health resources scarce in these places, but they also present problems of accessibility to health services that offer moderate to high-level technology.

Currently, tafenoquine is at an advanced stage of development and that has presented effectiveness in the prevention of relapse with just one dose, may be able to achieve the desired elimination of vivax malaria, but the fact that it is an 8-aminoquilonine means that it presents the same challenge in relation to safety for use with carriers of G6PDd, reinforcing the discussion about the need to detect the deficiency before starting treatment [6,19,43].

The current research estimated a high number of male individuals with G6PDd exposed to the use of PQ and demonstrates that this deficiency may have an important effect on the need for hospitalization, being associated also with a significant number of deaths in individuals infected by vivax malaria. The direct medical and non-medical costs arising from diagnosis and treatment of vivax malaria and from the severe adverse events resulting from the use of PQ are high for the health service. There is a predominance observed of costs related to complications that occur after the use of PQ, especially those linked to hospitalization, indicating that among those suffering from the deficiency the severe adverse events burden the public health system more than the diagnosis and treatment of vivax malaria. As regards hospitalization, although assistance provided in the ward has predominated, the cost arising from 6% of those with G6PDd who go to intensive care corresponded to 19% of the total hospitalization costs, demonstrating the importance of severely ill patients in the context being researched. The high cost of hospitalization of carriers of G6PDd was also confirmed in another context. An economic evaluation of the cost of this deficiency among Lebanese male newborns identified a cost of US$ 1,450.00 per hospitalization [44]. The average cost presented in Table 3 may be considered high in the Brazilian context, where the SUS budget directed towards surveillance, prevention and control of malaria in 2009, 2010 and 2011 was, respectively, US$ 12,115,577.89; US$ 9,622,159.09 and US$ 9,802,395.21 [45].

Although studies were not identified in the literature that evaluate the cost of G6PDd in the context under research, there are recent publications that indicate how urgent it is to carry out economic evaluations on this topic, highlighting especially the need to estimate the costs and effectiveness of incorporating rapid diagnostic tests (RDTs) to detect G6PDd. These should be used at the point of care before applying PQ [4,6,46], similar to what has been described in the Brazilian context in relation to the cost-effectiveness of RDTs for diagnosing malaria itself [30,47], helping in decision-making on the incorporation or not of this technology. Currently, two RDTs to detect G6PDd in field conditions (BinaxNOW® G6PD and CareStartTM G6PD) have been evaluated in malaria endemic areas [48,49]. However, there are still gaps in knowledge about the performance, stability and costs of RDTs [19].

The acute character of the complication under discussion, associated with the constitutional responsibility of the SUS to guarantee universal, complete and equitable assistance, means that the costs falling to the SUS constitute important data. They may be used to help in decision-making and in the development of other economic studies on the topic, especially ones aiming to evaluate the strategies for prevention of severe adverse events, based on the recommendation for diagnosis of G6PDd before using PQ [19]. In this ambit, it is fundamental that research be carried out in Latin America, aiming to study locally the cost and effectiveness of tests to detect G6PDd in field conditions to reduce the cost of hospitalization [46].

Economic evaluations developed in relation to malaria have become increasingly frequent, especially in Sub-Saharan Africa, in Asia and in South America. White et al. identified that the cost of diagnosing a case of malaria ranged from US$ 0.34 to US$ 9.34, the cost of treating a case of uncomplicated malaria from US$ 2.36 to US$ 23.65 and the cost of a case of complicated malaria from US$ 15.64 to US$ 137.87 [50].

This is the first study of the costs of G6PDd in Brazil, and the results presented here are based on sources of epidemiological and secondary economic data. It should be stressed that there is an absence of precise data concerning G6PDd for the whole of the Brazilian Amazon, which may differ because of the diversity in ethnic origin in the region. Furthermore, the data on prevalence of G6PDd, the proportion of individuals hospitalized, the proportion of deaths, the costs of hospital food and salaries for professionals were obtained in the state of Amazonas and generalized for the nine states that make up the Brazilian Amazon. This may have affected the validity of the cost estimates, although the simulation carried out in the analysis of sensitivity has demonstrated that the uncertainties mentioned did not substantially affect the total cost.

## Conclusion

Within the scope of the Brazilian strategy for controlling vivax malaria, G6PDd represents a serious problem for public health. It has particularly affected men of economically productive age who live in vulnerable communities. As the efforts directed towards controlling and eliminating malaria are based on using an 8-aminoquinoline, attention must be paid to the safety of those carrying G6PDd. The results presented constitute basic evidence in the discussion on the use of PQ without prior testing to detect G6PDd. It is recommended that a research agenda be adopted that will evaluate on a regional basis the costs and accuracy of RDTs to detect G6PDd, with the aim of checking if it is viable to incorporate these into the health system’s strategy.

## Additional file

Additional file 1:
**Sensitivity analysis of the costs of G6PDd among male individuals infected by** ***P. vivax*** **in the, Brazilian Amazon, 2009, 2010 and 2011.**

## Abbreviations

G6PD: Glucose-6-Phosphate Dehydrogenase
G6PDd: Glucose-6-Phosphate Dehydrogenase Deficiency
PQ: Primaquine
SUS: National Health System
NMCP: National Malaria Control Programme
RDTs: Rapid diagnostic tests
ICU: Intensive Care Unit

## References

[1] Cappellini MD, Fiorelli G (2008). Glucose-6-phosphate dehydrogenase deficiency. Lancet..

[2] Beutler E (2008). Glucose-6-phosphate dehydrogenase deficiency: a historical perspective. Blood..

[3] Minucci A, Moradkhani K, Jing M, Zuppi C, Giardina B, Capoluongo E (2012). Blood cells, molecules, and diseases glucose-6-phosphate dehydrogenase (G6PD) mutations database: Review of the “ old ” and update of the new mutations. Blood Cells Mol Dis..

[4] Von Seidlein L, Auburn S, Espino F, Shanks D, Cheng Q, McCarthy J (2013). Review of key knowledge gaps in glucose-6-phosphate dehydrogenase deficiency detection with regard to the safe clinical deployment of 8-aminoquinoline treatment regimens: a workshop report. Malar J..

[5] Monteiro WM, Franca GP, Melo GC, Queiroz ALM, Brito M, Peixoto HM (2014). Clinical complications of G6PD deficiency in Latin American and Caribbean populations: systematic review and implications for malaria elimination programmes. Malar J..

[6] Domingo GJ, Satyagraha AW, Anvikar A, Baird K, Bancone G, Bansil P (2013). G6PD testing in support of treatment and elimination of malaria: recommendations for evaluation of G6PD tests. Malar J..

[7] Howes RE, Piel FB, Patil AP, Nyangiri O, Gething PW, Dewi M (2012). G6PD deficiency prevalence and estimates of affected populations in malaria endemic countries: a geostatistical model-based map. PLoS Med..

[8] Ministério da Saúde. Sistema de informação de Vigilância Epidemiológica (SIVEP-Malária) [http://portalweb04.saude.gov.br/sivep_malaria/default.asp]

[9] Santana MS, Monteiro WM, Siqueira AM, Costa MF, Sampaio V, Lacerda MV (2013). Glucose-6-phosphate dehydrogenase deficient variants are associated with reduced susceptibility to malaria in the Brazilian Amazon. Trans R Soc Trop Med Hyg..

[10] Fundação de Medicina Tropical Dr. Heitor Vieira Dourado (FMT-HVD) (2011). Banco de dados - deficiência da glicose-6-fosfato-desidrogenase (2009–2011).

[11] Gething PW, Elyazar IRF, Moyes CL, Smith DL, Battle KE, Guerra CA (2012). A long neglected world malaria map: *Plasmodium vivax* endemicity in 2010. PLoS Negl Trop Dis..

[12] Ministério da Saúde (2010). Guia prático de tratamento da malária no Brasil.

[13] Wells TNC, Burrows JN, Baird JK (2010). Targeting the hypnozoite reservoir of *Plasmodium vivax*: the hidden obstacle to malaria elimination. Trends Parasitol..

[14] Alonso PL, Brown G, Arevalo-Herrera M, Binka F, Chitnis C, Collins F (2011). A research agenda for malaria eradication: drugs. PLoS Med..

[15] Lacerda MVG, Fragoso SCP, Alecrim MGC, Alexandre M, Magalhães BML, Siqueira AM (2012). Postmortem characterization of patients with clinical diagnosis of *Plasmodium vivax* malaria: to what extent does this parasite kill?. Clin Infect Dis..

[16] Superintendência do Desenvolvimento da Amazônia. Amazônia Legal [http://www.sudam.gov.br/demografia/50-amazonialegal]

[17] Ramos Júnior WM, Sardinha JF, Costa MR, Santana MS, Alecrim MG, Lacerda MV (2010). Clinical aspects of hemolysis in patients with *P. vivax* malaria treated with primaquine, in the Brazilian Amazon. Brazilian J Infect Dis.

[18] Fundação de Medicina Tropical Dr. Heitor Vieira Dourado (2014). Rotinas da Malária.

[19] Recht J, Ashley E, White N (2014). Safety of 8-Aminoquinoline Antimalarial Medicines.

[20] Ministério da Saúde. Sistema de Gerenciamento da Tabela de Procedimentos, Medicamentos e OPM do SUS [http://www.sudam.gov.br/demografia/50-amazonialegal]

[21] Ministério da Saúde. Banco de Preços em Saúde [http://aplicacao.saude.gov.br/bps/login.jsf]

[22] Instituto Brasileiro de Geografia e Estatística. Sistema Nacional de Índices de Preços ao Consumidor [http://www.ibge.gov.br/home/estatistica/indicadores/precos/inpc_ipca/defaultseriesHist.shtm]

[23] Ministério da Saúde (2005). Manual de diagnóstico laboratorial da malária.

[24] Macauley C (2005). Aggressive active case detection: a malaria control strategy based on the Brazilian model. Soc Sci Med..

[25] Oliveira MRF. Cost-effectiveness analysis of rapid test for the diagnosis of new malaria cases in twelve endemic municipalities of the State of Pará. *PhD thesis*. Universidade de São Paulo, Faculdade de Saúde Pública; 2009.

[26] Fernando SD, Karunaweera ND, Fernando WP, Attanayake N, Wickremasinghe R (2004). A cost analysis of the use of the rapid, whole-blood, immunochromatographic P.f/P.v assay for the diagnosis of *Plasmodium vivax* malaria in a rural area of Sri Lanka. Ann Trop Med Parasitol.

[27] Walker D, Kumaranayake L (2002). Allowing for differential timing in cost analyses: discounting and annualization. Health Policy Plan..

[28] Weinstein MC, Siegel JE, Gold MR, Kamlet MS, Russell LB (1996). Recommendations of the panel on cost-effectiveness in health and medicine. JAMA..

[29] Phillips M, Mills A, Dye C (1996). Directrices para el analisis del costo-eficacia de la lucha atnivectorial.

[30] Oliveira MRF, de Castro GA, Toscano CM (2010). Cost effectiveness of OptiMal® rapid diagnostic test for malaria in remote areas of the Amazon Region, Brazil. Malar J.

[31] World Health Organization - Regional Office for the Western Pacific (2005). Malaria light microscopy - creating a culture of quality.

[32] Ministério da Saúde. Sistema de Informação de Insumos Estratégicos [http://portalweb04.saude.gov.br/sies/]

[33] Fundação de Medicina Tropical Dr. Heitor Vieira Dourado (FMT-HVD) (2011). Custos com a nutrição hospitalar.

[34] Secretaria de Estado da Saúde do Amazonas (SES/AM) (2011). Tabela de vencimentos e gratificação de saúde.

[35] Oliveira-Ferreira J, Lacerda MVG, Brasil P, Ladislau JLB, Tauil PL, Daniel-Ribeiro CT (2010). Malaria in Brazil: an overview. Malar J..

[36] Ferreira ACS, Suárez-Mutis MC, Campos MR, de Castro CGSO (2011). Primary health care in municipalities at high risk for malaria. Rev Lat Am Enfermagem..

[37] Etim S, Ogbeche J (2014). Household responses to malaria: illness perception, cost implications and treatment-seeking behavior of mothers in Calabar, Nigeria. Malar J.

[38] Carter R, Mendis KN (2002). Evolutionary and historical aspects of the burden of malaria. Clin Microbiol Rev..

[39] Battle KE, Karhunen MS, Bhatt S, Gething PW, Howes RE, Golding N (2014). Geographical variation in *Plasmodium vivax* relapse. Malar J..

[40] Vitor-Silva S, Reyes-Lecca RC, Pinheiro TRA, Lacerda MVG (2009). Malaria is associated with poor school performance in an endemic area of the Brazilian Amazon. Malar J..

[41] Beutler E, Duparc S (2007). Glucose-6-phosphate dehydrogenase deficiency and antimalarial drug development. Am J Trop Med Hyg..

[42] Kondrashin A, Baranova AM, Ashley EA, Recht J, White NJ, Sergiev VP (2014). Mass primaquine treatment to eliminate vivax malaria: lessons from the past. Malar J..

[43] Llanos-Cuentas A, Lacerda MV, Rueangweerayut R, Krudsood S, Gupta SK, Kochar SK (2014). Tafenoquine plus chloroquine for the treatment and relapse prevention of *Plasmodium vivax* malaria (DETECTIVE): a multicentre, double-blind, randomised, phase 2b dose-selection study. Lancet..

[44] Khneisser I, Adib SM, Loiselet J, Megarbane A (2007). Cost-benefit analysis of G6PD screening in Lebanese newborn males. J Med Liban..

[45] Ministério do Planejamento, Orçamento e Gestão. Orçamento Federal [http://www.orcamentofederal.gov.br/orcamentos-anuais/orcamento-2010/orcamentos_anuais_view?anoOrc = 2010]

[46] Monteiro WM, Val FF, Siqueira AM, Franca GP, Sampaio VS, Melo GC (2014). G6PD deficiency in Latin America: systematic review on prevalence and variants. Mem Inst Oswaldo Cruz..

[47] Oliveira MRF, Giozza SP, Peixoto HM, Romero GAS (2012). Cost-effectiveness of diagnostic for malaria in extra-Amazon Region, Brazil. Malar J.

[48] Kim S, Nguon C, Guillard B, Duong S, Chy S, Sum S (2011). Performance of the CareStart^TM^ G6PD deficiency screening test, a point-of-care diagnostic for primaquine therapy screening. PLoS ONE..

[49] Osorio L, Carter N, Arthur P, Bancone G, Gopalan S, Gupta SK (2015). Performance of BinaxNOW G6PD deficiency point-of-care diagnostic in *P. vivax*-infected subjects. Am J Trop Med Hyg.

[50] White MT, Conteh L, Cibulskis R, Ghani AC (2011). Costs and cost-effectiveness of malaria control interventions - a systematic review. Malar J..

