# Supplementary material for: G6PD deficiency in male individuals infected by Plasmodium vivax malaria in the Brazilian Amazon: a cost study
Source: Malar J. 2015 Mar 24;14:126. doi: 10.1186/s12936-015-0647-x (PMC4381355; doi:10.1186/s12936-015-0647-x)
Supplement: Additional file 1: — Sensitivity analysis of the costs of G6PDd among male individuals infected by P. vivax in the, Brazilian Amazon, 2009, 2010 and 2011. [file 12936_2015_647_MOESM1_ESM.docx]

**Additional file 1: Table S1:** Sensitivity analysis of the costs of G6PDd among male individuals infected by *P. vivax* in the, Brazilian Amazon, 2009, 2010 and 2011.

| **Items and sources** | **Reference values considered in the sensitivity analysis: upper and lower variation** | | | | | |  |
| --- | --- | --- | --- | --- | --- | --- | --- |
| **A -** Diagnosis of vivax malaria | **2009 (US$)** |  | **2010 (US$)** |  | **2011(US$)** |  |  |
|  | **Lower limit** | **Upper Limit** | **Lower limit** | **Upper Limit** | **Lower limit** | **Upper Limit** |  |
| - Thick blood smear [24, 30] | 16,898.01 | 125,388.25 | 20,513.27 | 152,237.80 | 24,817.87 | 184,182.28 |  |
| - Microscope [25] | 39,458.46 | 59,187.68 | 46,101.73 | 69,152.60 | 46,928.89 | 70,393.34 |  |
| - Microscope maintenance [30] | 2,995.37 | 4,493.05 | 3,499.67 | 5,249.51 | 3,562.46 | 5,343.69 |  |
| - Health workers [31] | 140,867.89 | 211,301.12 | 171,158.04 | 256,736.22 | 212,008.17 | 318,010.34 |  |
| - Annual training (microscopist)[30] | 260,035.25 | 390,052.87 | 299,080.57 | 448,620.85 | 308,319.82 | 462,479.74 |  |
| - Total costs (A) | 460,254.97 | 790,422.97 | 540,353.28 | 931,996.97 | 595,637.21 | 1,040,409.38 |  |
| **B -** Drug treatment (*P. vivax)* |  |  |  |  |  |  |  |
| - Cost (B) Therapeutic scheme [32] | 1,857.92 | 1,857.92 | 2,638.56 | 2,638.56 | 1,863.65 | 1,863,65 |  |
| **C -** Assistance to **c**arriers of G6PDd treated with PQ |  |  |  |  |  |  |  |
| - Pre-admission tests [20, 24, 30] | 68,716.63 | 78,673.16 | 86,853.78 | 99,860.44 | 74,882.63 | 86,741.71 |  |
| - Medical appointments [20] | 66,271.36 | 66,271.36 | 83,681.82 | 83,681.82 | 72,047.90 | 72,047.90 |  |
| - Hospitalization [20] | 2,824,385.98 | 4,236,578.97 | 3,566,393.77 | 5,349,590.65 | 3,070,573.78 | 4,605,860.67 |  |
| - Hospital food [33] | 346,206.30 | 519,309.45 | 464,569.39 | 696,854.08 | 430,986.50 | 646,479.76 |  |
| - Health workers [34] | 113,461.31 | 170,191.96 | 150,805.15 | 226,207.73 | 140,196.44 | 210,294.66 |  |
| - Tests performed after hospitalization [20, 24, 31] | 46,462.18 | 56,219.58 | 58,751.07 | 71,497.60 | 50,684.84 | 62,306.75 |  |
| - Total costs (C) | 3,465,503.76 | 5,127,244.48 | 4,411,054.98 | 6,527,692.32 | 3,839,372.10 | 5,683,731.46 |  |
| **- Total costs (A+B+C)** | **3,927,616.65** | **5,919,525.37** | **4,954,046.82** | **7,462,327.86** | **4,436,872,96** | **6,726,004,49** |  |
